# Supplementary material for: Sorting at embryonic boundaries requires high heterotypic interfacial tension
Source: Nat Commun. 2017 Jul 31;8:157. doi: 10.1038/s41467-017-00146-x (PMC5537356; doi:10.1038/s41467-017-00146-x)
Supplement: Supplementary file 2 — Supplementary Software 1 [file 41467_2017_146_MOESM2_ESM.zip › PottsModel/SrcPottsModel/doc/model/class-use/Pixel.html]

Uses of Class model.Pixel


JavaScript is disabled on your browser.


Skip navigation links


- Overview
- Package
- Class
- Use
- Tree
- Deprecated
- Index
- Help

- Prev
- Next

- Frames
- No Frames

- All Classes

## Uses of Class model.Pixel

- Packages that use Pixel

  | Package | Description |
  | --- | --- |
  |  |  |
  | --- | --- |
  | engine |  |
  | gui |  |
  | model |  |
- - ### Uses of Pixel in engine

    Fields in engine declared as Pixel

    | Modifier and Type | Field and Description |
    | --- | --- |
    |  |  |
    | --- | --- |
    | `Pixel` | PottsEngine.StateVariables.`aPixelSwapSource` |
    | `Pixel` | PottsEngine.StateVariables.`aPixelSwapTarget` |
    | `Pixel[]` | PottsEngine.StateVariables.`aToRedraw` |
  - ### Uses of Pixel in gui

    Methods in gui with parameters of type Pixel

    | Modifier and Type | Method and Description |
    | --- | --- |
    |  |  |
    | --- | --- |
    | `void` | CellDisplay.`addOrRemovePixel(Pixel pPixel, boolean pIsAdded)` |
  - ### Uses of Pixel in model

    Methods in model that return Pixel

    | Modifier and Type | Method and Description |
    | --- | --- |
    |  |  |
    | --- | --- |
    | `Pixel[]` | Pixel.`copyTo(Pixel pTarget)` Move pixel from one cell to the other and make appropriate changes. |
    | `Pixel[]` | Pixel.`getAllNeighbors()` |
    | `Pixel[]` | SquarePositionManager.`getAllNeighbors(Pixel pPixel)` |
    | `Pixel[]` | IPositionManager.`getAllNeighbors(Pixel pPixel)` Returns an array of APixels whose length corresponds to the number of possible positions. |
    | `Pixel[]` | HexagonPositionManager.`getAllNeighbors(Pixel pPixel)` |
    | `Pixel[]` | SquarePositionManager.`getAllNeighbors(Pixel pPixel1, Pixel pPixel2)` |
    | `Pixel[]` | IPositionManager.`getAllNeighbors(Pixel pPixel1, Pixel pPixel2)` Get union of neighbors of pPixel1 and pPixel2. |
    | `Pixel[]` | HexagonPositionManager.`getAllNeighbors(Pixel pPixel1, Pixel pPixel2)` |
    | `Pixel[]` | Pixel.`getConnectedNeighbors()` |
    | `Pixel[]` | SquarePositionManager.`getConnectedNeighbors(Pixel pPixel)` |
    | `Pixel[]` | IPositionManager.`getConnectedNeighbors(Pixel pPixel)` |
    | `Pixel[]` | HexagonPositionManager.`getConnectedNeighbors(Pixel pPixel)` |
    | `Pixel` | SquarePositionManager.`getNeighborFromPosition(Pixel pPixel, IPositionManager.Position pPosition)` |
    | `Pixel` | IPositionManager.`getNeighborFromPosition(Pixel pPixel, IPositionManager.Position pPosition)` |
    | `Pixel` | HexagonPositionManager.`getNeighborFromPosition(Pixel pPixel, IPositionManager.Position pPosition)` |
    | `Pixel[]` | SquarePositionManager.`getPartialNeighbors(Pixel pPixel)` |
    | `Pixel[]` | IPositionManager.`getPartialNeighbors(Pixel pPixel)` |
    | `Pixel[]` | HexagonPositionManager.`getPartialNeighbors(Pixel pPixel)` |
    | `Pixel` | Lattice.`getPixel(int pX, int pY)` |
    | `Pixel` | Cell.`getPixel(int x, int y)` |
    | `Pixel` | Pixel.`getRandomNeighbor(java.util.Random r)` |
    | `Pixel` | Lattice.PixelIterator.`next()` |

    Methods in model that return types with arguments of type Pixel

    | Modifier and Type | Method and Description |
    | --- | --- |
    |  |  |
    | --- | --- |
    | `java.util.Iterator<Pixel>` | Lattice.`iterator()` |

    Methods in model with parameters of type Pixel

    | Modifier and Type | Method and Description |
    | --- | --- |
    |  |  |
    | --- | --- |
    | `void` | Cell.`addPixel(Pixel pPixel)` Add Pixel to Cell. |
    | `boolean` | SquarePositionManager.`areHorizontalNeighbours(Pixel pPixel1, Pixel pPixel2)` |
    | `boolean` | IPositionManager.`areHorizontalNeighbours(Pixel pPixel1, Pixel pPixel2)` |
    | `boolean` | HexagonPositionManager.`areHorizontalNeighbours(Pixel pPixel1, Pixel pPixel2)` |
    | `static boolean` | HexagonPositionManager.`areNeighbors(Pixel pPixel1, Pixel pPixel2)` |
    | `boolean` | SquarePositionManager.`areNeighbours(Pixel pPixel1, Pixel pPixel2)` |
    | `boolean` | IPositionManager.`areNeighbours(Pixel pPixel1, Pixel pPixel2)` |
    | `boolean` | HexagonPositionManager.`areNeighbours(Pixel pPixel1, Pixel pPixel2)` |
    | `static boolean` | Pixel.`areSwappable(Pixel pSource, Pixel pTarget)` Check if resulting swap will not produce a disconnected pixel. |
    | `boolean` | SquarePositionManager.`areVerticalNeighbours(Pixel pPixel1, Pixel pPixel2)` |
    | `boolean` | IPositionManager.`areVerticalNeighbours(Pixel pPixel1, Pixel pPixel2)` |
    | `boolean` | HexagonPositionManager.`areVerticalNeighbours(Pixel pPixel1, Pixel pPixel2)` |
    | `double` | IEnergyFunction.`computeEnergyLocally(Lattice aLattice, Pixel pSource, Pixel pTarget)` Computes the energy of the two pixels and their neighbours. |
    | `double` | IEnergyFunction.`computeEnergyLocallyIfSwapped(Lattice aLattice, Pixel pSource, Pixel pTarget)` |
    | `Pixel[]` | Pixel.`copyTo(Pixel pTarget)` Move pixel from one cell to the other and make appropriate changes. |
    | `Pixel[]` | SquarePositionManager.`getAllNeighbors(Pixel pPixel)` |
    | `Pixel[]` | IPositionManager.`getAllNeighbors(Pixel pPixel)` Returns an array of APixels whose length corresponds to the number of possible positions. |
    | `Pixel[]` | HexagonPositionManager.`getAllNeighbors(Pixel pPixel)` |
    | `Pixel[]` | SquarePositionManager.`getAllNeighbors(Pixel pPixel1, Pixel pPixel2)` |
    | `Pixel[]` | IPositionManager.`getAllNeighbors(Pixel pPixel1, Pixel pPixel2)` Get union of neighbors of pPixel1 and pPixel2. |
    | `Pixel[]` | HexagonPositionManager.`getAllNeighbors(Pixel pPixel1, Pixel pPixel2)` |
    | `Pixel[]` | SquarePositionManager.`getConnectedNeighbors(Pixel pPixel)` |
    | `Pixel[]` | IPositionManager.`getConnectedNeighbors(Pixel pPixel)` |
    | `Pixel[]` | HexagonPositionManager.`getConnectedNeighbors(Pixel pPixel)` |
    | `static double[]` | AreaEnergyFunction.`getCurrentCellAreas(Pixel s, Pixel t)` |
    | `static double[]` | PerimeterEnergyFunction.`getCurrentCellPerimeters(Pixel pSource, Pixel pTarget)` |
    | `double` | PixelInteractionEnergyFunction.`getDeltaEnergy(Pixel s, Pixel t)` |
    | `double` | PerimeterEnergyFunction.`getDeltaEnergy(Pixel s, Pixel t)` |
    | `double` | InterfacialTensionEnergyFunction.`getDeltaEnergy(Pixel s, Pixel t)` |
    | `double` | EnergyManager.`getDeltaEnergy(Pixel s, Pixel t)` |
    | `double` | EnergyFunction.`getDeltaEnergy(Pixel s, Pixel t)` |
    | `double` | AreaEnergyFunction.`getDeltaEnergy(Pixel s, Pixel t)` |
    | `static double[]` | AreaEnergyFunction.`getFutureCellAreas(Pixel s, Pixel t)` |
    | `static double[]` | PerimeterEnergyFunction.`getFutureCellPerimeters(Pixel pSource, Pixel pTarget)` |
    | `double` | IEnergyFunction.`getLocalPixelInteractions(Pixel pPixel)` Computes the boundary energy around a single pixel. |
    | `Pixel` | SquarePositionManager.`getNeighborFromPosition(Pixel pPixel, IPositionManager.Position pPosition)` |
    | `Pixel` | IPositionManager.`getNeighborFromPosition(Pixel pPixel, IPositionManager.Position pPosition)` |
    | `Pixel` | HexagonPositionManager.`getNeighborFromPosition(Pixel pPixel, IPositionManager.Position pPosition)` |
    | `Pixel[]` | SquarePositionManager.`getPartialNeighbors(Pixel pPixel)` |
    | `Pixel[]` | IPositionManager.`getPartialNeighbors(Pixel pPixel)` |
    | `Pixel[]` | HexagonPositionManager.`getPartialNeighbors(Pixel pPixel)` |
    | `double` | Pixel.`getPixelInteraction(Pixel pNeighbor)` Checks whether pixels are neighbors and then calculates interaction based on cell type. |
    | `static double` | Pixel.`getPixelInteraction(Pixel pPixel1, Pixel pPixel2)` Checks whether pixels are neighbors and then calculates interaction based on cell type. |
    | `boolean` | Pixel.`isInSameCell(Pixel pTarget)` |
    | `boolean` | Pixel.`isNeighborWith(Pixel pPixel)` |
    | `void` | Cell.`removePixel(Pixel pPixel)` Removes pixel from cell and readjusts center of mass, area and perimeter if it's not medium. |
    | `void` | Cell.`updateParameters(Pixel pPixel, boolean pIsAdded)` Call after a pixel has been added or removed from a cell |

    Constructors in model with parameters of type Pixel

    Constructor and Description

    `Pixel(Pixel pPixel)`

    `Pixel(Pixel pPixel,
    Cell pC)`

Skip navigation links


- Overview
- Package
- Class
- Use
- Tree
- Deprecated
- Index
- Help

- Prev
- Next

- Frames
- No Frames

- All Classes
